# Feasibility and validation of trans-valvular flow derived by four-dimensional flow cardiovascular magnetic resonance imaging in patients with atrial fibrillation

**DOI:** 10.12688/wellcomeopenres.16655.2

**Published:** 2021-05-18

**Authors:** Mark T Mills, Ciaran Grafton-Clarke, Gareth Williams, Rebecca C Gosling, Abdulaziz Al Baraikan, Andreas L Kyriacou, Paul D Morris, Julian P Gunn, Peter P Swoboda, Eylem Levelt, Vasiliki Tsampasian, Rob J van der Geest, Andrew J Swift, John P Greenwood, Sven Plein, Vass Vassiliou, Pankaj Garg

**Affiliations:** 1 Department of Infection, Immunity & Cardiovascular Disease, University of Sheffield, Sheffield, UK; 2 Norwich Medical School, University of East Anglia, Norwich, UK; 3 Leeds Institute of Cardiovascular and Metabolic Medicine, University of Leeds, Leeds, UK; 4 Department of Radiology, Leiden University Medical Center, Leiden, The Netherlands

**Keywords:** 4D flow CMR, Haemodynamics, Flow quantification, Validation, Atrial fibrillation

## Abstract

**Background**: Four-dimensional (4D) flow cardiovascular magnetic resonance imaging (MRI) is an emerging technique used for intra-cardiac blood flow assessment. The role of 4D flow cardiovascular MRI in the assessment of trans-valvular flow in patients with atrial fibrillation (AF) has not previously been assessed. The purpose of this study was to assess the feasibility, image quality, and internal validity of 4D flow cardiovascular MRI in the quantification of trans-valvular flow in patients with AF.

**Methods**: Patients with AF and healthy controls in sinus rhythm underwent cardiovascular MRI, including 4D flow studies. Quality assurance checks were done on the raw data and streamlines. Consistency was investigated by trans-valvular flow assessment between the mitral valve (MV) and the aortic valve (AV).

**Results**: Eight patients with AF (88% male, mean age 62±13 years, mean heart rate (HR) 83±16 beats per minute (bpm)) were included and compared with ten healthy controls (70% male, mean age 41±20 years, mean HR 68.5±9 bpm). All scans were of either good quality with minimal blurring artefacts, or excellent quality with no artefacts. No significant bias was observed between the AV and MV stroke volumes in either healthy controls (–4.8, 95% CI –15.64 to 6.04; P=0.34) or in patients with AF (1.64, 95% CI –4.7 to 7.94; P=0.56). A significant correlation was demonstrated between MV and AV stroke volumes in both healthy controls (r=0.87, 95% CI 0.52 to 0.97; P=0.001) and in AF patients (r=0.82, 95% CI 0.26 to 0.97; P=0.01).

**Conclusions**: In patients with AF, 4D flow cardiovascular MRI is feasible with good image quality, allowing for quantification of trans-valvular flow.

## Introduction

Atrial fibrillation (AF) is the most common sustained cardiac rhythm disturbance in adults ^1^. Recent data suggest the prevalence of AF is between 2% and 4% worldwide ^1^, with this figure expected to rise ^2, 3^. Whilst AF can occur in individuals without other cardiovascular comorbidities, it is frequently associated with cardiac disease, including heart failure (with reduced and preserved ejection fraction), valvular disease, and ischaemic heart disease. Cardiovascular magnetic resonance imaging (MRI) is assuming a prominent role in the management of patients with AF, both in the assessment of atrial substrate in the pre-ablation setting ^4^ and in those with associated cardiovascular conditions with concomitant AF ^5^.

Four-dimensional flow (4D flow) cardiovascular MRI is an emerging technique used for intra-cardiac blood flow assessment in the research and clinical setting. Previous studies have demonstrated accurate and reliable assessment of valvular flow and regurgitation quantification using retrospective valve tracking methods ^6, 7^. Furthermore, 4D flow-derived valvular flow assessment circumvents issues with Doppler-based echocardiography measurements and two-dimensional phase-contrast (PC) acquisition cardiovascular MRI ^8^. As with most cardiovascular MRI techniques, 4D flow traditionally requires cardiac gating using electrocardiography (ECG) in order to depict the various cardiac structures and movements. Cardiac rhythms with high R-R interval variability such as AF can result in significant image quality degradation and challenging image analyses. As a result, the feasibility, precision, and reliability of valvular flow quantification in patients with AF have not previously been assessed.

We hypothesised that 4D flow cardiovascular MRI is feasible in patients with AF and can accurately and consistently quantify valvular flow. The aims of this study were to (i) assess the feasibility of acquiring 4D flow MRI in patients with AF and (ii) investigate the consistency and reliability of retrospective valve tracking in the quantification of aortic and mitral valvular flow, comparing patients with AF, against healthy controls.

## Methods

### Ethical considerations

The study protocol was approved by the National Research Ethics Service (12/YH/0169) in the UK. The study was approved by the local ethics committee and complied with the Declaration of Helsinki. All patients gave written informed consent immediately prior to MRI examinations.

### Study design and population

This was an observational cross-sectional study. In order to assess for internal validity, a healthy cohort was used as a comparator. For the study group, we recruited eight patients with persistent AF from the cardiology department at Leeds Teaching Hospitals NHS Trust. Patients were approached during either hospital admission or outpatient clinic review. Inclusion criteria included patients over the age of 18 years old with AF and a stable heart rate between 70 to 110 beats per minute; the only exclusion criterion was contraindication to MRI. For the healthy control group, another cohort of volunteers in normal sinus rhythm with no previous cardiovascular disorders were recruited. Controls were approached through an appeal for healthy volunteers via the University of Leeds and were not matched to AF cases. As the purpose of the study was to determine feasibility of 4D flow cardiovascular MRI in AF, power calculations were not performed to determine sample size.

***Cardiovascular MRI.*** All patients underwent cardiovascular MRI on a 1.5 Tesla system (Ingenia, Philips, Best, The Netherlands), with a 28-channel flexible torso coil, with digitisation of the MR signal in the receiver coil, at the University of Leeds between January 2014 and December 2017. As previously described ^9, 10^, the cardiovascular MRI protocol included baseline survey, cine imaging (vertical long axis, horizontal long axis, short-axis contiguous left-ventricle volume stack) acquired using balanced steady-state free precession in a single slice breath-hold sequence, and whole heart 4D flow acquired using a fast field echo pulse sequence (echo-planar imaging [EPI] based with sensitivity encoding [SENSE] acceleration) with retrospective ECG triggering. Typical 4D image parameters were as follows: EPI acceleration factor of 5 and SENSE factor of 2, flip angle 10°, velocity encoding 150 cm/s, field of view 400 mm, echo time 3.5 ms, repetition time 10 ms, 90% partial k-space coverage in phase-encoding directions, number of signal averages 1; images were acquired during free breathing; the number of slices was 35–40, with a temporal resolution of 40 ms; the reconstructed number of phases was 30 ^10^.

***Image analysis.*** Image analysis was performed offline using MASS software (Version 2018EXP, Leiden University Medical Centre, Leiden, The Netherlands). Similar types of analysis can be carried out by open source software packages like FourFlow. All images were analysed by an imaging cardiologist (PG) with more than five years’ experience working with 4D flow cardiovascular MRI. Left ventricular volumes and ejection fraction were calculated from cine images using standard methods ^11^: endocardial and epicardial contours were traced on the left ventricular and right ventricular cine stack at end-systole and end-diastole to determine end systolic volume, end diastolic volume, stroke volume, ejection fraction and left ventricular mass. Volumes were indexed to body surface area. For each 4D flow data set, a visual quality assessment on the phase contrast and magnitude images was performed to determine image quality and presence of artefact, and scored on a 4-point scale ^9, 10^: 0, excellent quality without artefacts; 1, good quality with mild blurring artefacts; 2, moderate quality with moderate blurring artefacts; 3, poor quality with severe artefacts leading to non-evaluable data.

**Figure 1.**
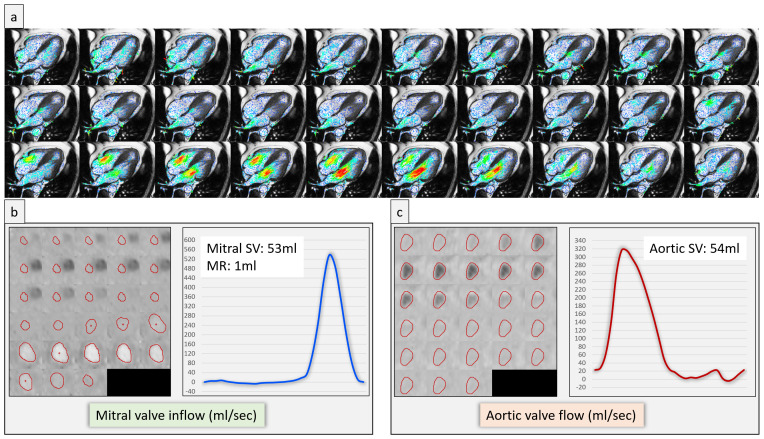
A case example of a patient in atrial fibrillation demonstrating two-dimensional velocity vectors superimposed over cine images ( **a**), and an example of segmentation of valvular flow contours on the phase contrast multiplanar reconstruction alongside mitral valve stroke volumes (SV) ( **b**) and aortic valve SV ( **c**).

***Flow and velocity measurement.*** Contour segmentation was performed manually. 4D flow assessments were performed using validated techniques including retrospective valve tracking, with measurement planes located perpendicular to the inflow direction on two- and four-chamber cines ^10, 12^ ( Figure 1). Background velocity corrections were performed from the velocity sampled in the myocardium and phase unwrapping was undertaken on source images if aliasing occurred within in the area of interest, as per our previous work ^10, 13– 16^. Trans-valvular forward flow and regurgitant flow were measured over the entire cardiac cycle for both the mitral and the aortic valve from the reconstructed dynamic phase-contrast plane. Trans-valvular stroke volume across the aortic valve (AV) and mitral valve (MV) was calculated as the difference between absolute forward flow and regurgitant flow through each valve ( Figure 1). In patients with AF, trans-valvular flow was measured as the average of velocities over 10 to 12 heart beats in order to remove beat-to-beat variability. Through-plane myocardial motion was accounted for in the above calculations.

***Statistical analysis*** Statistical analysis was performed using MedCalc Version 19.6.3. Continuous variables are expressed as mean ± standard deviation and categorical variables as numbers and percentages. Data was treated as normally distributed. Demographic comparisons were performed using an independent samples t-test. Student t-test was used to compare paired data. Bland-Altman plots were used to visually assess the agreement between methods and investigate absolute bias. Correlation assessment between AV and MV transvalvular stroke volumes was performed using Pearson’s correlation coefficient test. Applicable tests were two-tailed; P values < 0.05 were deemed significant.

## Results

### Patient characteristics

Eight patients with AF were included and compared with ten healthy controls in sinus rhythm ^17^. Baseline demographics are detailed in Table 1. Seven (88%) of the AF patients were male and the mean age was 62 ± 13 years. Age, body mass index, and heart rate were significantly higher in patients with AF than healthy controls.

**Table 1.** Baseline demographics.

	Healthy controls (n=10)	AF patients (n=8)	P-Value
**Age** (years)	41±20	62±13	0.02
**Sex** (female)	3	1	0.4
**Weight** (kg)	72±12	94±16	<0.01
**Height** (cm)	170±8	174±10	0.32
**Body mass index** (kg/m ^2^)	25±2	31±5	<0.01
**Heart rate** (bpm)	68.5±9	83±16	0.03
**Smoker** (n)	0	3	0.02
**Hypertension** (n)	0	3	0.02
**Hypercholesterolaemia** (n)	0	1	0.24
**Diabetes mellitus** (n)	0	1	0.24

### Image quality assessment

All scans were of either good quality with minimal blurring artefacts, or excellent quality with no artefacts (grade 1 and 0, respectively).

### Baseline cardiovascular MR scan parameters

Baseline cardiovascular MR scan parameters are summarised in Table 2. Left ventricular end-systolic volumes (LV-ESV) were significantly higher in patients with AF compared to healthy controls (LV-ESV AF: 133 ± 42 ml, control: 59 ± 15 ml; P < 0.01). Left ventricular stroke volume (LV-SV) and ejection fraction (LV-EF) were significantly lower in AF patients (LV-SV AF: 69 ± 20 ml, control: 97 ± 29 ml; P = 0.03; LV-EF AF: 35 ± 9%, control: 62 ± 6%; P < 0.01). AV stroke volumes were significantly lower in AF patients (AF: 51 ±13 ml, control: 78 ±30 ml; P=0.04); MV stroke volume was also lower in those with AF, but this did not meet statistical significance (AF: 53 ±12 ml, control: 73 ±26 ml; P=0.06). Patients with AF had a mitral regurgitant volume of 6 ±6 ml; none of the healthy controls had mitral regurgitation.

**Table 2.** Cardiovascular MRI parameters.

	Health controls (n=10)	AF patients (n=8)	P-Value
**LV-EDV (ml)**	155±39	202±39	0.02
**LV-ESV (ml)**	59±15	133±42	<0.01
**LV-SV (ml)**	97±29	69±20	0.03
**LV-EF (%)**	62±6	35±9	<0.01
**LV-EDM (g)**	94±24	129±30	0.01
**LV-ESM (g)**	96±25	134±30	0.01
**LV-EDVi (ml/m ^2^)**	84±16	95±20	0.18
**LV-ESVi (ml/m ^2^)**	32±7	63±21	<0.01
**LV-SVi (ml/m ^2^)**	52±12	32±9	<0.01
**LV-EDMi (g/m ^2^)**	50±10	61±13	0.06
**MV SV (ml)**	73±26	53±12	0.06
**MR volume (ml)**	0±0	6±6	<0.01
**AV SV (ml)**	78±30	51±13	0.04

Abbreviations: AV, aortic valve; EDM, end-diastolic mass; EDV, end-diastolic volume; EF, ejection fraction; ESM, end-systolic mass; ESV, end-systolic volume; LV, left ventricular; MV, mitral valve; MR, mitral regurgitant; SV, stroke volume. The suffix i represents indexed values to body surface area.

### Consistency in 4D flow derived valvular stroke volumes

**Figure 2.**
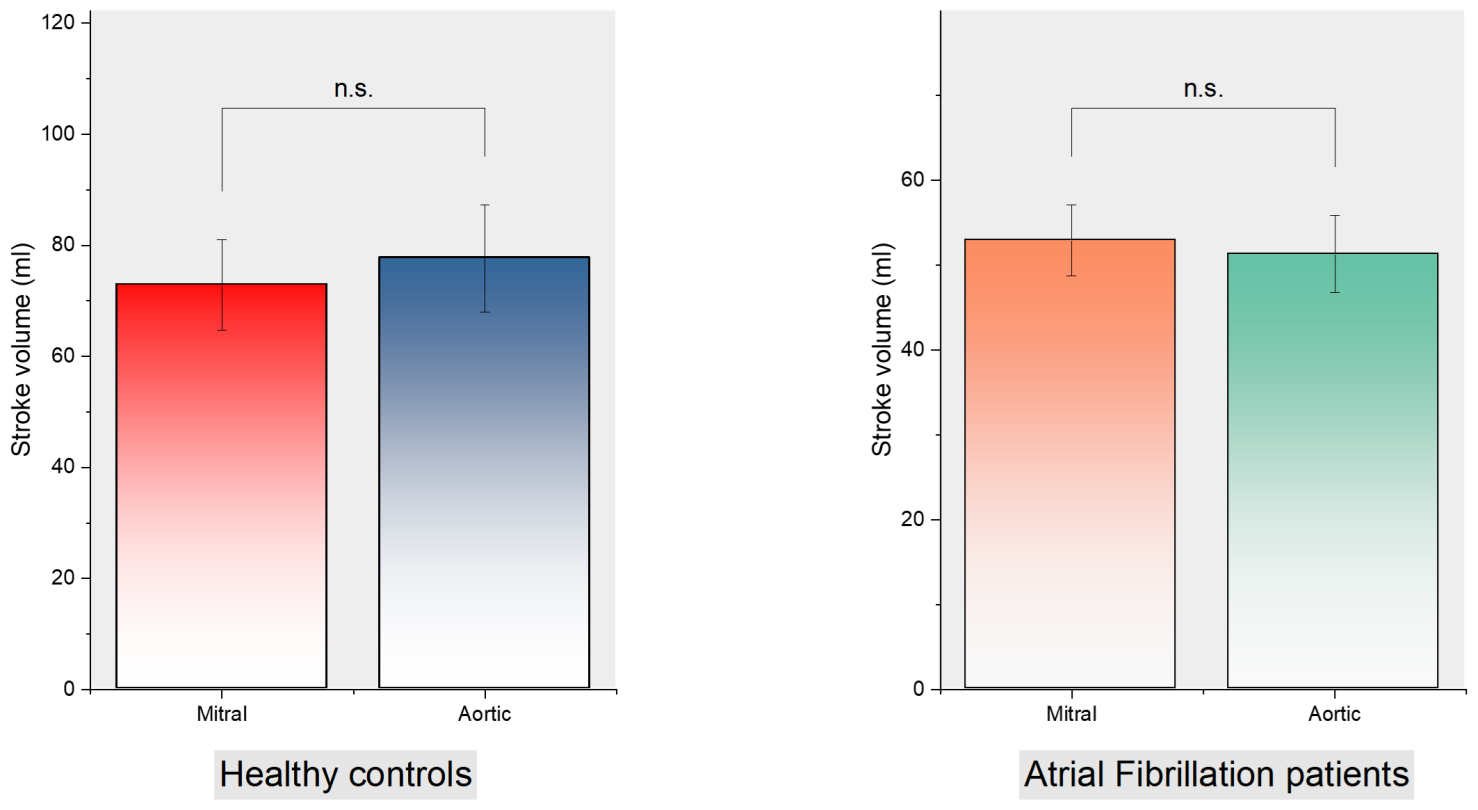
Mitral and aortic valve stroke volumes in healthy controls and patients with atrial fibrillation.

**Figure 3.**
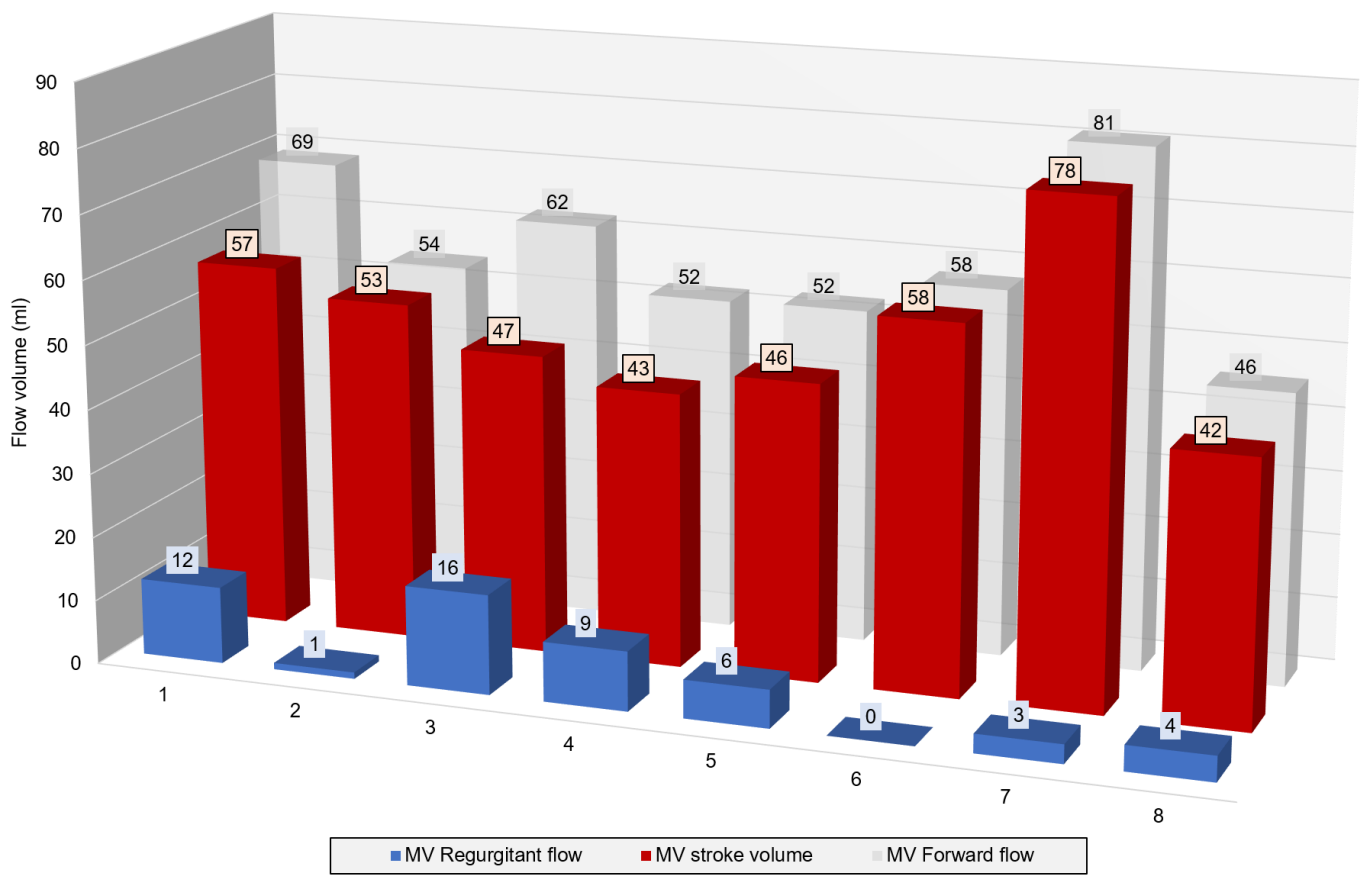
Mitral valve (MV) forward flow, regurgitant flow and stroke volume for individual patients with atrial fibrillation.

**Figure 4.**
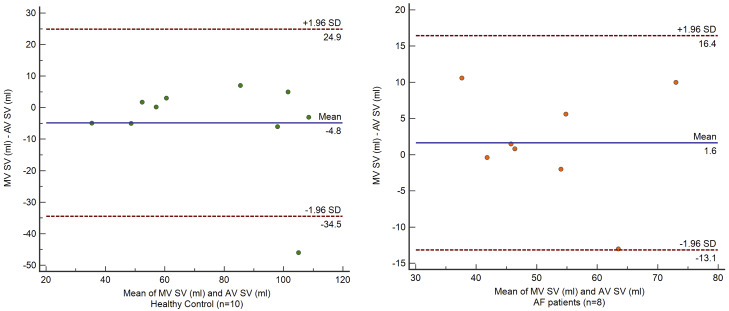
Bland-Altman plot of the mean of mitral valve (MV) and aortic valve (AV) stroke volumes (SV) in healthy control and patients with atrial fibrillation.

**Figure 5.**
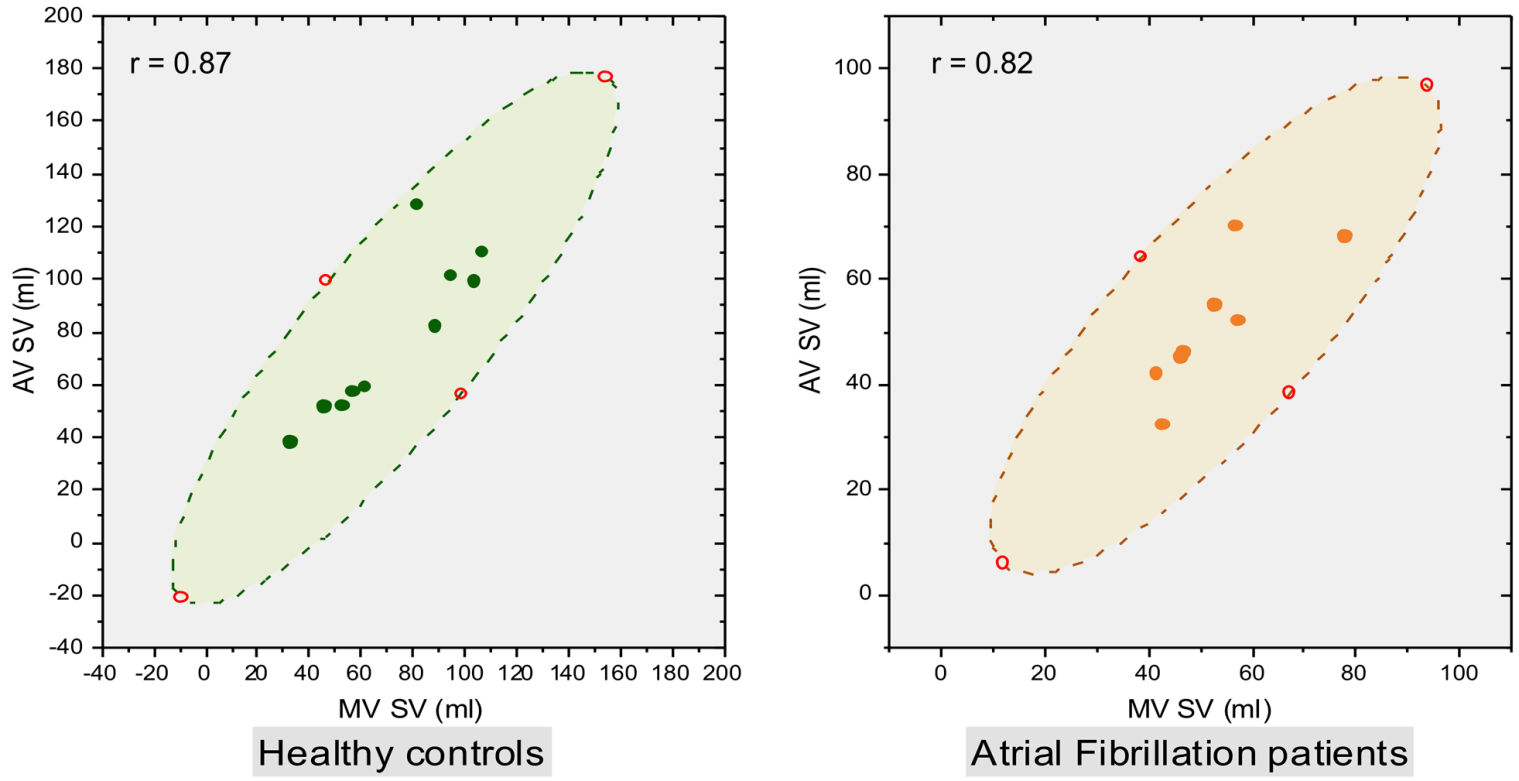
Pearson correlation (r) between mitral valve (MV) stroke volume (SV) and aortic valve (AV) SV in healthy controls and patients with atrial fibrillation. The shaded areas represent 95% confidence intervals.

There was no significant difference in MV and AV stroke volumes in healthy controls (72.85 ± 25.87 ml vs. 77.65 ± 30.45 ml respectively; P=0.34) or in patients with AF (52.92 ± 11.8 ml vs. 51.29 ± 12.86 ml; P=0.56) ( Figure 2). MV forward flow, regurgitant flow and stroke volume for individual AF patients are detailed in Figure 3. In both AF patients and controls, the mean MV and AV stroke volumes were compared with the difference between MV and AV stroke volumes by Bland-Altman analysis ( Figure 4). No significant bias was observed, although bias was higher in healthy controls (–4.8, 95% CI –15.64 to 6.04; P=0.34) than in patients with AF (1.64, 95% CI –4.7 to 7.94; P=0.56). There was a strong correlation between MV and AV stroke volumes in healthy controls (Pearson’s correlation r = 0.87, 95% CI 0.52 to 0.97; P=0.0011) and AF patients (r = 0.82, 95% CI 0.26 to 0.97; P=0.0134) ( Figure 5).

## Discussion

This study investigated the feasibility of performing 4D flow cardiovascular MRI in patients with AF for the accurate assessment of trans-valvular flow. We demonstrated that 4D flow cardiovascular MRI is feasible in patients with rate-controlled AF, and that it allows accurate quantification of trans-valvular flow and regurgitation in patients with AF. 

### Known applications of 4D flow cardiovascular MRI in AF

Previous research applications of 4D flow cardiovascular MRI in patients with AF are limited and include the assessment of left atrial (LA) flow and stasis ^18– 23^, the assessment of LA and LV function following cardioversion ^18, 24^, and the effect of AF on aortic flow ^25^. Kim *et al.* observed that LV stroke volume and components of LV flow (including delayed ejection, retained inflow, and residual volume) were significantly different between patients with paroxysmal AF (in sinus rhythm at the time of 4D flow cardiovascular MRI) and healthy controls ^26^, suggesting that the substrate leading to AF in itself may lead to LV flow impairment, rather than the rhythm disturbance alone.

### Quantitative assessment of trans-valvular stroke volume

No previous 4D flow cardiovascular MRI studies have examined the assessment of trans-valvular flow in patients with AF. In order to ensure the appropriate application of 4D flow cardiovascular MRI in patients with AF in both the research and clinical settings, the validity, accuracy, and reliability of trans-valvular flow measurements are fundamental. Our study demonstrates that there is no significant bias between AV and MV stroke volumes in patients with AF, similar to healthy controls, and found a strong correlation between MV and AV stroke volumes in both groups. As no reference method for MV or AV trans-valvular stroke volumes exists in patients with AF, we used a mean of both measurements when examining for agreement between measurements ( Figure 4). In echocardiography studies, the optimal mean number of beats required to estimate AV flow and cardiac output in AF is 13 beats (with a range of 4 to 17) to achieve a variability of less than 2%, compared to a mean of 4 beats in sinus rhythm ^27, 28^. Similarly, we measured trans-valvular flow as the average of velocities over many heart beats, in order to remove beat-to-beat variability. 4D flow MRI may therefore have an advantage to quantify transvalvular flow in atrial fibrillation. We noticed that the LV stroke volume from cine segmentation were higher in both the healthy and AF cohorts when compared to respective mitral and aortic flows. We suspect this is mainly due to variation of basal slice segmentation on cine, as it is well known that slight variations in basal segmentation of endocardium can result in significant differences in stroke volume.

### Quantitative assessment of mitral regurgitation

Mitral regurgitation remains a common clinical entity in patients with AF. The superiority of cardiovascular MRI in the quantitative evaluation of mitral regurgitant volume in comparison with 2-dimensional echocardiography is well established ^29, 30^. Our data highlights the precision with which 4D flow cardiovascular MRI is able to calculate mitral regurgitant volume in AF, within 3–4 ml ( Figure 3). This allows the severity of mitral regurgitation to be graded with incredible accuracy compared with echocardiography. These findings warrant investigation in larger cohorts.

### Limitations

The number of patients with AF included in this study was low. However, the key findings were based on intra-group comparisons showing internal validity. Secondly, we recruited patients with rate-controlled AF and in stable conditions. The results of this study cannot therefore be extrapolated to patients in AF with a ventricular rate greater than 110bpm. Third, patients underwent a single scan only, without longitudinal follow-up. Given previous evidence of physiological variability over time in patients with AF ^31^, a temporal assessment would be beneficial.

## Conclusion

Whole-heart, 4D flow cardiovascular MRI is feasible in patients with AF and allows reliable quantification of trans-valvular flow and regurgitation.

## Data availability

### Underlying data

Access to the raw images of patients is not permitted since specialised post-processing imaging-based solutions can identify the study patients in the future.

Harvard Dataverse: Feasibility and validation of trans-valvular flow derived by four-dimensional flow cardiovascular magnetic resonance imaging in patients with atrial fibrillation. https://doi.org/10.7910/DVN/YHDLIT ^17^.

This project contains the following underlying data:

- Data for repository.tab (spreadsheet demographic and outcome variables)

Data are available under the terms of the Creative Commons Zero "No rights reserved" data waiver (CC0 1.0 Public domain dedication).

## Consent

All patients gave written informed consent prior to MRI examinations.
