# Supplementary material for: Feasibility and validation of trans-valvular flow derived by four-dimensional flow cardiovascular magnetic resonance imaging in patients with atrial fibrillation
Source: Wellcome Open Res. 2021 May 18;6:73. Originally published 2021 Mar 31. [Version 2] doi: 10.12688/wellcomeopenres.16655.2 (PMC8150120; doi:10.12688/wellcomeopenres.16655.2)
Supplement: Peer review history

# Peer review: Reviewer response for version 2

**DOI:** 10.21956/wellcomeopenres.18674.r43954

**Published:** 2021-05-25

**Authors:** Victoria M. Stoll

**Affiliations:** 1 Institute of Cardiovascular Sciences, University of Birmingham, Birmingham, UK

The authors have fully addressed my concerns and I am happy to approve the article without reservation.

Is the work clearly and accurately presented and does it cite the current literature?

Yes

If applicable, is the statistical analysis and its interpretation appropriate?

Yes

Are all the source data underlying the results available to ensure full reproducibility?

Partly

Is the study design appropriate and is the work technically sound?

Yes

Are the conclusions drawn adequately supported by the results?

Yes

Are sufficient details of methods and analysis provided to allow replication by others?

Yes

Reviewer Expertise:

4D flow, congenital heart disease, cardiac MRI

I confirm that I have read this submission and believe that I have an appropriate level of expertise to confirm that it is of an acceptable scientific standard.

# Peer review: Reviewer response for version 2

**DOI:** 10.21956/wellcomeopenres.18674.r43955

**Published:** 2021-05-19

**Authors:** Jordi Broncano

**Affiliations:** 1 Cardiothoracic Imaging Section, Health Time, Hospital de la Cruz Roja and San Juan de Dios, Cordoba, Spain

No new comments to make. Appreciate the work done by the authors.

Is the work clearly and accurately presented and does it cite the current literature?

Yes

If applicable, is the statistical analysis and its interpretation appropriate?

Yes

Are all the source data underlying the results available to ensure full reproducibility?

Yes

Is the study design appropriate and is the work technically sound?

Yes

Are the conclusions drawn adequately supported by the results?

Yes

Are sufficient details of methods and analysis provided to allow replication by others?

Yes

Reviewer Expertise:

Cardiac MRI

I confirm that I have read this submission and believe that I have an appropriate level of expertise to confirm that it is of an acceptable scientific standard.

# Peer review: Reviewer response for version 1

**DOI:** 10.21956/wellcomeopenres.18364.r43358

**Published:** 2021-05-14

**Authors:** Victoria M. Stoll

**Affiliations:** 1 Institute of Cardiovascular Sciences, University of Birmingham, Birmingham, UK

This article aims to assess the use of 4D flow in patients with atrial fibrillation. In particular it looks at the trans-valvular flow across the mitral compared to aortic valve. 

 10 healthy controls and 8 patients with atrial fibrillation were enrolled in the study. All scans were deemed to be of sufficient quality for analysis. The study found good correlations between MV and AV flow and no significant bias in these stroke volumes.

 I only have very minor suggestions for the manuscript:

- Given there was no statistical difference in LV end diastolic volume when indexed values are used I would remove the comment about non-indexed end diastolic volumes being significantly different. 
- Do you have any echo data on these patients? The very low EF is quite striking and I wondered how accurate that is or whether it is a reflection of the difficultly of imaging these patients with MR cine images.
- When presenting the data for the flow across the mitral valve then aortic valve it would be easier if you kept the order the same between all figures - figure 1 has Ao valve, then mitral whereas all other figures have the other order. Additionally fig 2 shows the bars as MV then Ao valve but the figure legend says Aortic valve first. Also on Figure 2 the bar graph for AF patients has a stroke volume 1 as the y axis label - I presume this should just be stroke volume?
- Table 1 has a tiny typo of 'health' rather than 'healthy'.
- Figure 3 - can all data be presented to the same number of significant figures please - I would assume whole numbers are most appropriate as I doubt the techniques can be as accurate as to two decimal places. 
- Otherwise this is a soundly conducted study showing that 4D flow parameters can be measured in patients with AF. Did any patients undergo repeat scans to look at stability over time? If not it would be worth adding this lack of temporal assessment to the limitations section as it would be interesting to see if AF patients have more or less variability then healthy controls (see Stoll *et al.*, 2018 ^1^ who found likely physiological variability to be important over time).

Is the work clearly and accurately presented and does it cite the current literature?

Yes

If applicable, is the statistical analysis and its interpretation appropriate?

Yes

Are all the source data underlying the results available to ensure full reproducibility?

Partly

Is the study design appropriate and is the work technically sound?

Yes

Are the conclusions drawn adequately supported by the results?

Yes

Are sufficient details of methods and analysis provided to allow replication by others?

Yes

Reviewer Expertise:

4D flow, congenital heart disease, cardiac MRI

I confirm that I have read this submission and believe that I have an appropriate level of expertise to confirm that it is of an acceptable scientific standard, however I have significant reservations, as outlined above.

## References

[1] : Test-retest variability of left ventricular 4D flow cardiovascular magnetic resonance measurements in healthy subjects. *Journal of Cardiovascular Magnetic Resonance*.2018;20(1) : 10.1186/s12968-018-0432-4 10.1186/s12968-018-0432-4 29499706PMC5833126

# Response

**Published:** 2021-05-17

**Authors:** Mark Mills

**Affiliations:** University of Sheffield, UK

*This article aims to assess the use of 4D flow in patients with atrial fibrillation. In particular it looks at the trans-valvular flow across the mitral compared to aortic valve.*

**

*10 healthy controls and 8 patients with atrial fibrillation were enrolled in the study. All scans were deemed to be of sufficient quality for analysis. The study found good correlations between MV and AV flow and no significant bias in these stroke volumes.*

**

*I only have very minor suggestions for the manuscript:*

*Given there was no statistical difference in LV end diastolic volume when indexed values are used I would remove the comment about non-indexed end diastolic volumes being significantly different.*

**Response:** We thank the reviewer for their feedback and for giving us the opportunity to improve our manuscript. We have removed the comment regarding end-diastolic volumes being different from the text.

*Do you have any echo data on these patients? The very low EF is quite striking and I wondered how accurate that is or whether it is a reflection of the difficultly of imaging these patients with MR cine images.*

**Response** **:** We thank the reviewer for this comment. Unfortunately, we do not have echo data on these patients.

*When presenting the data for the flow across the mitral valve then aortic valve it would be easier if you kept the order the same between all figures - figure 1 has Ao valve, then mitral whereas all other figures have the other order. Additionally fig 2 shows the bars as MV then Ao valve but the figure legend says Aortic valve first. Also on Figure 2 the bar graph for AF patients has a stroke volume 1 as the y axis label - I presume this should just be stroke volume?*

**Response:** We thank the reviewer for their feedback.

 In figure 1, we have switched figure b and c around so that mitral valve is first, and aortic valve second (consistent with subsequent figures). The figure 1 legend has also been amended to reflect this change.

 The legend to figure 2 has been amended to state “Mitral and aortic valve stroke volumes […]”.

 The y axis on Figure 2 (AF patients) has been corrected to “stroke volume”.

*Table 1 has a tiny typo of 'health' rather than 'healthy'.*

**Response:** Thank you for identifying this. We have modified it.

*Figure 3 - can all data be presented to the same number of significant figures please - I would assume whole numbers are most appropriate as I doubt the techniques can be as accurate as to two decimal places.*

**Response:** We thank the reviewer for pointing this out. We have changed this to whole numbers.

*Otherwise this is a soundly conducted study showing that 4D flow parameters can be measured in patients with AF. Did any patients undergo repeat scans to look at stability over time? If not it would be worth adding this lack of temporal assessment to the limitations section as it would be interesting to see if AF patients have more or less variability then healthy controls (see Stoll et al., 20181 who found likely physiological variability to be important over time).*

**Response:** Thank you for your comment. Patients did not undergo repeat scans. We have added the following sentence to the limitations section:

 “Third, patients underwent a single scan only, without longitudinal follow-up. Given previous evidence of physiological variability over time in patients with AF (Stoll et al., 2018), a temporal assessment would be beneficial.”

 This new reference has also been added to the reference list (reference 31).

 31. Stoll VM, Loudon M, Eriksson J, et al.: Test-retest variability of left ventricular 4D flow cardiovascular magnetic resonance measurements in healthy subjects. *J Cardiovasc Magn Reson*. 2018;20(1):15. 10.1186/s12968-018-0432-4

# Peer review: Reviewer response for version 1

**DOI:** 10.21956/wellcomeopenres.18364.r43357

**Published:** 2021-04-30

**Authors:** Jordi Broncano

**Affiliations:** 1 Cardiothoracic Imaging Section, Health Time, Hospital de la Cruz Roja and San Juan de Dios, Cordoba, Spain

With this original paper, the authors provide further insight on the feasibility of 4D flow analysis in a complex cohort for obtaining cardiac images such as in patients with atrial fibrillation. 

**Introduction**:

 Well balanced with clear identification of the clinical problem and the main purpose of the study. 

**Materials & methods and results:**

 Well presented and detailed. Image analysis methodology clearly displayed. Graphics and results help to explain the main objective of the study. 

 Minor comment: The AV and MV abbreviations are defined in the abstract but should be defined in the main text.

**Discussion and conclusion**: 

 Well-conducted. Comprehensive exposure of the limitations. Conclusion well presented. 

**Tables**:

 Well balanced with clear information and adequate regarding the objectives stated in the study.

**Images**:

 Figure 1a. The only comment I have with this figure is that the large amount of images in it makes difficult to visualize the great quality of the 4D flow images. I would suggest to reduce the number of images included in figure 1a.

Is the work clearly and accurately presented and does it cite the current literature?

Yes

If applicable, is the statistical analysis and its interpretation appropriate?

Yes

Are all the source data underlying the results available to ensure full reproducibility?

Yes

Is the study design appropriate and is the work technically sound?

Yes

Are the conclusions drawn adequately supported by the results?

Yes

Are sufficient details of methods and analysis provided to allow replication by others?

Yes

Reviewer Expertise:

Cardiac MRI

I confirm that I have read this submission and believe that I have an appropriate level of expertise to confirm that it is of an acceptable scientific standard.

# Response

**Published:** 2021-05-17

**Authors:** Mark Mills

**Affiliations:** University of Sheffield, UK

*With this original paper, the authors provide further insight on the feasibility of 4D flow analysis in a complex cohort for obtaining cardiac images such as in patients with atrial fibrillation.*

**Response: **We thank the insightful reviewer for their positive feedback.

*Introduction:*

*Well balanced with clear identification of the clinical problem and the main purpose of the study.*

**Response:** We thank the reviewer for their feedback.

*Materials & methods and results:*

*Well presented and detailed. Image analysis methodology clearly displayed. Graphics and results help to explain the main objective of the study.*

**Response:** We thank the reviewer for their feedback.

*Minor comment: The AV and MV abbreviations are defined in the abstract but should be defined in the main text.*

**Response:** Thank you for highlighting this. This has now been modified.

*Discussion and conclusion:*

*Well-conducted. Comprehensive exposure of the limitations. Conclusion well presented.*

**Response:** We thank the reviewer for their feedback.

*Tables:*

*Well balanced with clear information and adequate regarding the objectives stated in the study.*

**Response:** We thank the reviewer for their feedback.

*Images:*

*Figure 1a. The only comment I have with this figure is that the large amount of images in it makes difficult to visualize the great quality of the 4D flow images. I would suggest to reduce the number of images included in figure 1a.*

**Response: **We thank the reviewer for this comment. The reason for inclusion of all the images is to show how 4D flow 2D vectors performed throughout the cardiac cycle. ****Hence, we have not made changes to this figure. Nevertheless, we have addressed comments from reviewer 2 regarding all figures.
